# Correlates of HIV status awareness among older adults in Uganda: results from a nationally representative survey

**DOI:** 10.1186/s12889-018-6027-z

**Published:** 2018-09-17

**Authors:** Anne M. Nabukenya, Joseph K. B. Matovu

**Affiliations:** 1 MakSPH-CDC Fellowship Program, Makerere University School of Public Health, Kampala, Uganda; 2 Department of Community Health and Behavioral Sciences, Makerere University School of Public Health, P.O. Box 7072, Kampala, Uganda

**Keywords:** Older adults, HIV/ AIDS, HCT

## Abstract

**Background:**

Recent evidence suggests that HIV prevalence is generally higher among older than younger persons. However, few studies have explored issues regarding HIV testing and awareness of HIV status among older persons. We explored the correlates of HIV status awareness among older adults (aged 45+ years) in Uganda.

**Methods:**

This paper is based on secondary analysis of existing data on persons aged between 45 and 59 years from a nationally representative Uganda AIDS Indicator Survey which was conducted between February and September 2011. Records on the socio-demographics and HIV/AIDS-specific indicators for 2472 persons were extracted for analysis. Individuals were considered to be aware of their HIV status if they reported that they had tested and received their HIV test results within the past 12 months. Data analyses were done using the sample survey procedures to take into account the sampling structure of the data. Odds ratios were used to quantify the associations between receipt of HIV test results and potential factors.

**Results:**

Of the 2472 respondents, 48% had ever tested and received their HIV test results while 23% tested and received their HIV results in the past 12 months or already knew that they are HIV positive. Individuals with the following characteristics had higher odds of being aware of their HIV status: being female (adjusted Odds Ratio (AOR) = 1.26; 95% CI: (1.04, 1.53), having high comprehensive knowledge of HIV/AIDS (AOR = 1.28; 95% CI: 1.04, 1.58), having attended secondary school education (AOR = 2.10; 95% CI: 1.47, 2.99) and engagement in high risk sexual behaviors (AOR = 1.53; 95% CI: (1.11, 2.10). A high level of stigma (holding at least three stigmatizing attitudes toward people living with HIV) was negatively correlated with awareness of HIV status (AOR =0.60; 95% CI: (0.45, 0.78).

**Conclusion:**

Less than a quarter of older Ugandans are aware of their current HIV status. High levels of stigma and low comprehensive knowledge of HIV/AIDS remained critical barriers to HIV testing and awareness of HIV status. These findings suggest a need for innovative HIV testing strategies to increase HIV status awareness among older adults in Uganda.

## Background

Majority of individuals aged above 50 years are still sexually active and are, therefore, at risk of HIV acquisition. However, this age group is rarely targeted in HIV prevention interventions [1]. Whereas individuals in this age group perceive themselves to be at lower risk of HIV infection [2], globally, new HIV infections among persons aged above 50 years are still high and HIV prevalence is still increasing [1]. As anti-retroviral therapy (ART) is rolled out, more HIV positive individuals are living longer thus furthering the ageing of the epidemic. Thus, individuals who reach the age of 50 years while still HIV negative still interact with equally old potential sexual partners who are HIV positive.

HIV voluntary counseling and testing (HCT) is widely accepted as the cornerstone of HIV prevention and care programs in many countries because of its multiple benefits [3–8]. In addition to being a main entry point to treatment, HCT is seen as a key component of public health efforts to lower HIV incidence [9]. Benefits of HCT for individual clients include the fact that it empowers the uninfected person to protect him or herself from becoming infected with HIV, and assists infected persons to protect others and to live positively. Studies show that people who are aware of their HIV sero-status are more likely to practice safer sex [10, 11] and, if HIV-infected, awareness of HIV status offers them the opportunity for early HIV treatment initiation and to protect others from the risk of HIV infection. HIV testing uptake has grown rapidly with the expansion of ART in many countries including Uganda. In Uganda, home-based HIV testing and mobile camps have been used to promote HIV testing [12]. However, trends in HCT uptake rates among persons aged 45 years and older have rarely been reported.

Several factors may be responsible for an individual’s decision to know his or her HIV status. Previous studies have shown that more educated adults are more likely to test for HIV than others [13, 14]. Studies have also shown that HIV-related stigma may be a major deterrent in a person’s decision to seek HIV testing [9]. Stigma for HIV/AIDS oftentimes leads to late testing, diagnosis, and reluctance to seek services [15–17]. Evidence shows that people who feel stigmatized are reluctant to engage in HIV prevention, testing, and treatment largely because of the fear of the negative social consequences should their HIV test results turn out to be HIV-positive [9]. In addition to stigma, the reluctance of older adults to seek an HIV test is also influenced by lack of HCT promotional messages that are specific to them. Many HCT promotional messages have mainly targeted younger persons. Further, health workers often under-estimate the risk of HIV acquisition in this age group and this in turn reinforces HIV-k8related stigma [18]. Knowledge of HIV prevention information has also been shown to be associated with HIV testing in some studies [19, 20].

Unfortunately, little is still known about the extent to which older persons are aware of their HIV status, and this has implications for timely linkage to appropriate HIV prevention, care and treatment services. Thus, knowledge of the factors affecting HIV testing and receipt of HIV test results will help to inform policymakers and program implementers about the issues that need to be addressed to improve HIV programming for this older population. In this study, we explored the factors affecting the awareness of HIV status among older adults in Uganda using a nationally representative sample.

## Methods

### Study design and population

The paper is based on secondary analysis of existing data on persons aged between 45 and 59 years from the Uganda AIDS Indicator Survey (UAIS) conducted between February and September 2011 [21]. The 2011 UAIS was a nationally representative, cross-sectional, household sero-survey from which data of total 2472 persons aged 45 to 59 years was extracted.

### Sampling and data extraction procedures

The UAIS methodology including the data collection procedures have been previously reported [21]. In brief, the survey used a two-stage, stratified sampling design to provide national estimates from nine sero-survey regions across the country. In the first stage, a total of 470 enumeration areas (EAs) were selected, and from each area, a random sample of 25 households was selected for a total of 11,750 households. Data were collected on household and individual characteristics. Blood samples were obtained from consenting adults for HIV serology. This analysis is based on selected socio-demographic and HIV/AIDS-specific indicators of 2472 sexually active adults aged between 45 and 59 years, for whom complete HIV testing data were available. The selected indicators included prior HIV counseling and testing (HCT) experiences, knowledge of HCT service sites, self-perception of HIV risk, history of sexually transmitted infections (including STIs in the past 12 months), sexual risky behaviors (i.e., multiple sexual partners and condom use at last sex), comprehensive knowledge of HIV/AIDS, age, education level, wealth index, employment status and HIV stigma. These factors were selected because they have been associated with uptake of HCT in previous studies [12–19].

### Measurement of variables

The primary outcome of this analysis was awareness of HIV status among older Ugandans that were interviewed as part of the 2011 UAIS. In the survey, participants were asked ‘*Have you ever been tested to see if you have/do not have the virus that causes AIDS?*’ and if they responded in the affirmative, a follow-up question was asked: ‘*When was the last time you were tested?*’ Individuals were asked if they received the result of their last test and if not, whether or not they were already aware of their HIV status at the time of interview. All those who tested and reported receiving their HIV test results in the past 12 months (regardless of whether it was positive or negative) and those who reported that they were already aware of their HIV positive status at the time of interview were considered to be aware of their HIV status.

For the knowledge of HIV/AIDS, a respondent was considered to have comprehensive knowledge if she or he stated that (1) people can reduce the chances of acquiring HIV by using a condom every time before sex, (2) people can reduce the chance of acquiring HIV by having sex with just one partner who is not infected and who has no other partner; (3) people cannot acquire HIV from mosquito bites; (4) people cannot acquire HIV from sharing food with a person who has it, and that (5) a healthy looking person can have HIV.

Wealth index scores were constructed using household asset data via principal component analysis and categorized into quintiles as described in UDHS 2011 report [21]. An HIV stigma index was created from seven questions reflecting both accepting and negative attitudes about people living with HIV/AIDS. In the survey, the respondents were asked to state yes or no to the following questions: *“Would you buy fresh vegetables from a vendor who has the virus that causes AIDS?”; “If a female teacher has the virus that causes AIDS but is not sick, should she be allowed to continue teaching in school?”; “If a family member got infected with the virus that causes AIDS, would you be willing to care for her or him in your own household?”;* “*If a member of your family got infected with the virus that causes AIDS, would you want it to remain a secret?”*. The respondents were also asked to state if they agree or disagree with the following statements: “*People with the AIDS virus should be ashamed of themselves” and “people with the AIDS virus should be blamed for bringing the disease into the community*”. The responses were appropriately summated and categorized into 0 (*“No stigma”), 1–2 (“Low stigma”) and above 3 (“High stigma”).* All measures were based on self-reports.

### Data analysis

Data analyses were done using the sample survey procedures to take into account the sampling structure (stratification, sample weighting, and clustering) and sub-setting. The primary variable for our analysis was “awareness of HIV status”. All categorical variables were summarized using frequencies and proportions. At bivariate analysis, the associations between awareness of the current HIV status and the potential factors were assessed using the Rao-Scott adjusted Chi-square tests. Only factors with the Rao-Scott adjusted Chi-square *p*-values of <=0.20 were included in a multivariable logistic regression model. However, age group and sex were included in the multivariable model as a priori confounders. A factor was excluded from the multivariable logistic model when the change in the adjusted log-likelihood ratio was not significant on its addition or removal. For collinear factors such as involvement in high risk sex and having multiple sexual partners, only one was included in the multivariable model. All analyses were done using STATA version 12. Analysis took into account survey sampling weights using a suite of survey commands in STAT v12.

## Results

### Sample characteristics

Data were extracted from a sample of 2543 respondents aged 45–59 years, who were sexually active in the past 12 months prior to the survey. Table 1 shows the socio-demographic characteristics of the study sample. Of these, 2472 (97%) had complete data on HIV testing within the 12 months. Of the 2472 respondents, 59% were males; 50% were aged above 49 years; 91% were married or cohabiting; and 88% of the participants were living in rural areas. Further, 23 % had no formal education but majority were employed and notably in the informal agricultural sector.

**Table 1:** Sample demographic characteristics and bivariate analysis of demographic factors

Characteristic	Total	Tested in last 12 months		
	N (%)	n (%)	χ^2^	*p*-value
Sex			0.831	0.363
Male	1446 (58.5)	325 (22.5)		
Female	1026 (41.5)	246 (24.1)		
Age group			3.993	0.019
45–49	1234 (49.9)	280 (22.8)		
50–54	785 (31.8)	200 (26.0)		
55–59	453 (18.3)	91 (19.2)		
Education level			7.332	< 0.001
None	560 (22.7)	108 (18.6)		
Primary	1436 (58.1)	316 (22.1)		
Secondary	326 (13.2)	95 (29.5)		
Higher	150 (6.1)	52 (35.4)		
Region			4.077	< 0.001
Central 1	213 (8.6)	48 (22.2)		
Central 2	239 (9.7)	67 (27.8)		
Kampala	130 (5.3)	38 (25.6)		
East Central	278 (11.2)	60 (22.5)		
Mid-Eastern	332 (13.4)	71 (22.3)		
North East	220 (8.9)	60 (28.0)		
West Nile	278 (11.2)	50 (18.9)		
Mid Northern	254 (10.3)	88 (36.4)		
South Western	260 (10.5)	45 (17.1)		
Mid-Western	268 (10.8)	44 (15.7)		
Residence			0.090	0.764
Urban	293 (11.9)	73 (24.0)		
Rural	2179 (88.1)	498 (23.0)		
Religion			0.774	0.523
Catholic	1116 (45.1)	254 (22.9)		
Protestant	843 (34.1)	182 (21.4)		
Other Christian	211 (8.5)	58 (26.7)		
Muslim	279 (11.3)	74 (26.7)		
Other/none	23 (0.9)	3 (25.0)		
Marital status			1.052	0.350
Married/living together	2237 (90.5)	517 (23.1)		
Never married	23 (0.9)	3 (10.9)		
Divorced/separated	212 (8.6)	51 (24.8)		

Almost all participants (2448; 99%) had ever heard of AIDS and identified different preventive measures to HIV acquisition. However, only 35% had comprehensive knowledge of HIV/AIDS. Eleven percent reported to have been involved in high risk sex (sex with a non-cohabiting or non-married partner) while 14% reported multiple sexual partnerships in the past 12 months. At least 65% had some form of stigmatizing attitudes with about 10% classified as having high stigmatizing attitudes (Table 2). Eleven percent reported to have had an STI within the previous 12 months while only 20% of the respondents perceived themselves to be at high risk of acquiring HIV.

**Table 2:** Bivariate analysis of socio-economic and clinical factors associated with awareness of HIV status

Characteristic	Total	Tested in last 12 months		
	N (%)	n (%)	χ^2^	*p*-value
Wealth index			2.423	0.047
Poorest	497 (20.1)	109 (22.7)		
Poorer	497 (20.1)	102 (21.0)		
Middle	517 (20.9)	102 (19.3)		
Richer	535 (21.6)	144 (27.4)		
Richest	426 (17.2)	114 (25.3)		
Current employment status			1.723	0.190
Not employed	268 (10.8)	51 (19.5)		
Employed	2204 (89.2)	520 (23.5)		
Comprehensive knowledge of HIV/AIDS			15.642	< 0.001
No	1612 (65.2)	330 (20.3)		
Yes	860 (34.8)	241 (28.3)		
Perceived risk for HIV acquisition			1.288	0.276
High	491 (19.9)	131 (26.1)		
Low	1555 (62.9)	350 (22.6)		
Don’t know	426 (17.2)	90 (21.5)		
Had a STI in past 12 months			3.119	0.045
No	2132 (86.2)	471 (22.1)		
Yes	279 (11.3)	80 (28.8)		
Don’t know	61 (2.5)	20 (31.0)		
Feelings of Stigma			37.138	< 0.001
No stigma	565 (23.1)	159 (28.2)		
Low (1–2 stigmatizing attitudes/practices)	1101 (45.0)	267 (24.2)		
High (3–7 stigmatizing attitudes/practices)	761 (31.1)	145 (19.0)		
Don’t know	21 (0.9)	0 (0.0)		
Involved in high risk sex in past 12 months			12.837	< 0.001
No	2200 (89.0)	484 (21.9)		
Yes	272 (11.0)	87 (33.3)		
No. of partners in last 12 months			10.839	0.001
1	2123 (85.9)	467 (21.6)		
2+	349 (14.1)	104 (32.2)		

### HIV testing in the past 12 months and the associated factors

Of the 2472, 48% had ever tested and received their HIV test results and 23% tested and received their HIV results in the past 12 months or knew their HIV positive status. Of those who have ever tested, only 48% had a recent test in the past 12 months. At bivariate analysis, sex, age group, education level, religion, wealth index, sexual behavior, having had an STI in the past 12 months, stigma and low comprehensive knowledge of HIV/AIDS were significantly associated with awareness of HIV status in the past 12 months (Tables 1 and 2). Individuals reporting high levels of stigma (20% versus 29% among individuals with no stigma; *p*-value < 0.001) and those with limited comprehensive knowledge of HIV/AIDS (20% versus 28%; *p*-value < 0.001) were less likely to be aware of their HIV status. The proportions of those reporting to have received HCT in the past 12 months increased with increasing education levels and increasing wealth index. Marital status, place of residence (rural versus urban) and employment status were not associated with awareness of one’s HIV status.

Results from multivariable analysis are presented in Table 3. Education level, female (sex), risky sexual behavior, stigma and comprehensive knowledge of HIV/AIDS were associated with awareness of HIV status. Women are more likely to be aware of their HIV status than men (adjusted Odds Ratio (AOR) = 1.26; 95% CI: 1.04, 1.53). Individuals who had comprehensive knowledge of HIV/AIDS (AOR = 1.28; 95% CI: 1.04, 1.58) and those reporting multiple sexual partners (i.e. high risk sexual behavior) had higher odds of being aware of their HIV status than their counterparts (AOR = 1.53; 95% CI: 1.11, 2.10) (Table 3). There was an increasing trend towards higher odds of HIV status awareness with increasing levels of education. In particular, individuals with post-secondary education had more than twice the odds of being aware of their HIV status than individuals with no formal education (AOR = 2.10; 95% CI: 1.47, 2.99). A high level of stigma (holding at least three stigmatizing attitudes toward people living with HIV) was negatively correlated with awareness of HIV status (AOR =0.60; 95% CI: 0.45, 0.78).

**Table 3:** Multivariable logistic model for correlates of awareness of HIV status among older adults in Uganda

Characteristic	N	% tested	AOR (95% CI)	*p*-value
Sex				
Male	1446	22.5	1.00	
Female	1026	24.1	1.26 (1.04, 1.53)	0.019
Age group				
45–49	1234	22.8	1.00	
50–54	785	26.0	1.16 (0.97, 1.40)	0.110
55–59	453	19.2	0.90 (0.71, 1.15)	0.427
Education level				
None	560	18.6	1.00	
Primary	1436	22.1	1.59 (1.23, 2.05)	< 0.001
Secondary	326	29.5	2.10 (1.47, 2.99)	< 0.001
Higher	150	35.4	2.00 (1.22, 3.28)	0.006
Wealth index				
Poorest	497	22.7	1.00	
Poorer	497	21.0	0.74 (0.53, 1.04)	0.080
Middle	517	19.3	0.66 (0.49, 0.90)	0.009
Richer	535	27.4	0.85 (0.62, 1.17)	0.309
Richest	426	25.3	0.76 (0.54, 1.07)	0.119
Comprehensive knowledge of HIV/AIDS				
No	1612	20.3	1.00	
Yes	860	28.3	1.28 (1.04, 1.58)	0.019
Had a STI in past 12 months				
No	2132	22.1	1.00	
Yes	279	28.8	1.34 (1.00, 1.80)	0.047
Don’t know	61	31.0	1.54 (0.87, 2.73)	0.136
Stigma index				
No stigma	579	28.2	1.00	
Low (1–2 stigmatizing attitudes/practices)	1337	24.2	0.84 (0.67, 1.05)	0.132
High (3–7 stigmatizing attitudes/practices)	256	19.0	0.60 (0.45, 0.78)	< 0.001
Involvement in risk behavior				
No	2123	21.6	1.00	
Yes	349	32.2	1.53 (1.11, 2.10)	0.009
Perceived risk for HIV acquisition				
High	491	26.1	1.00	
Low	1555	22.6	0.72 (0.45, 1.10)	0.219
Don’t know	426	21.5	0.69 (0.41, 1.12)	0.201

In general, wealth index had no significant effect on one’s awareness of their current HIV status at multivariate analysis. Whereas, individuals in households in the third wealth quintile had lower odds of being aware of their HIV status, there was no consistency in upward trend or down trend to confirm the effect of wealth index on testing for HIV.

## Discussion

In this study, we explored the correlates of HIV status awareness among Ugandans aged 45 years and above. Our findings show that less than a quarter of older Ugandans are aware of their current HIV status. High levels of stigma, lack comprehensive knowledge of HIV/AIDS and low self-perception of HIVAIDS acquisition remain critical barriers to awareness of HIV status among this group of people. In a related study by Kenyon [22] these same factors were found to influence awareness of HIV positive status.

There is a high level of stigma particularly among older persons. At the age of 50, they may already face isolation due to illness or loss of family and friends [23]. The stigma negatively affects the quality of life, self-image and sexual behaviors and may prevent them from seeking HIV care and disclosing their HIV status. Studies show that home-based HCT can reduce HIV testing-related stigma and improve HIV testing uptake [24] suggesting that home-based HCT can help to increase the proportion older persons who are aware of their HIV status. Similarly, in our Ugandan setting, such door to door or routine community clinics can be an alternative approach to HIV testing for older persons as seen elsewhere. Thus, our findings call for a need to utilize alternative HIV testing approaches that can minimize stigma associated with HIV testing while improving HIV risk-perception among older Ugandans. Hopefully, this will increase the proportion of older persons testing for HIV; and hence the proportion of older persons who are aware of their HIV status.

Our study has some limitations. The main limitation being that all our study variables were self-reported. It is possible that participants were motivated to underreport stigma because of social desirability biases. We also did not account for a proportion of individuals who tested for HIV as a must as opposed to voluntary testing. Also, our findings cannot be generalized to people 60 years and older, because we considered those between 45 and 59 years. Nonetheless, our study is exceptional in that it uses a national survey data which was representative of all regions in Uganda. The other strength is that the analysis has explored correlates of HIV status awareness among older people who usually receive less attention in HIV programming yet they are still sexually active.

## Conclusion

Overall, our findings provide evidence that awareness of HIV status among older adults is still undesirable. Whereas women are more likely to test than men, barriers such as stigma and low comprehension of HIV information cut across both sexes. Our findings highlight the need to develop innovative HIV counseling and testing strategies to promote and encourage older adults to routinely access HCT services. Potential approaches include door-to-door HCT services, mass media communication targeting the older adults and taking an opportunity to counsel and test for HIV among individuals seeking healthcare services for other diseases.

## Abbreviations

AIDS: Acquired Immune Deficiency Syndrome
AOR: Adjusted Odds Ratio
ART: Antiretroviral Therapy
CI: Confidence Interval
HCT: HIV Counseling and Testing
HIV: Human Immunodeficiency Virus
STI: Sexually Transmitted Infection
UAIS: Uganda AIDS Indicator Survey
UDHS: Uganda Demographic and Health Survey
